# Study of the Interfacial Transition Zone Characteristics of Geopolymer and Conventional Concretes

**DOI:** 10.3390/gels8020105

**Published:** 2022-02-09

**Authors:** Hani Alanazi

**Affiliations:** Department of Civil and Environmental Engineering, College of Engineering, Majmaah University, AL-Majmaah 11952, Saudi Arabia; hm.alanazi@mu.edu.sa

**Keywords:** interfacial transition zone, geopolymer concrete, traditional concrete, nanoindentation, microstructure

## Abstract

The properties and performance of geopolymer at different length scales have been intensively studied, but only limited studies on geopolymer have investigated the zone located between paste and aggregates, which is called the interfacial transition zone (ITZ). The microstructure of ITZ and its nanomechanical properties in geopolymer concrete are examined in this study. Fly ash-based geopolymer has great potential to be an alternative to traditional concrete. To this end, scanning electron microscopy (SEM) and nanoindentation tests were performed to investigate the microstructural characteristics and nanomechanical properties of the ITZ, and the results were compared with the ITZ of traditional concrete. Results show that traditional concrete demonstrated a weak ITZ with pores and microcracks, while the geopolymer concrete microstructure did not present weak ITZs in the vicinity of aggregates. More pores and crack were observed in the ITZ in traditional concrete. Further, a considerable amount of fly ash particles, that appear to be unreacted or partially reacted in the matrix phase, was observed. Based on the nanoindentation results, 58% of the microstructure is composed of unreacted or partially reacted fly ash particles. The results of nano- and microscale tests will enhance the understanding of how concrete behaves and performs at large scales.

## 1. Introduction

The macroscale properties of a composite is greatly influenced by its interfaces. The interfacial transition zone (ITZ), which is known as the most important interface in concrete, is located between cement paste and aggregate in traditional concrete [1,2]. In the vicinity of each aggregate, the formation of ITZ takes place with a thickness of up to 50 μm [3]. In this zone, a higher amount of calcium hydroxide (CH) and ettringite can be found [4]. The ITZs have higher porosity compared to the matrix [4,5]. More micro cracks are also generated in the ITZs due to the fluctuation in strength between ITZs and aggregate and paste [6,7]. In addition, aggressive agents can penetrate into the concrete body via porous ITZs, which could lead to a large reduction in the mechanical properties and durability of concrete [1,8]. Thus, the ITZ is the weakest zone in the concrete, which makes it important, because the overall composite performance greatly depends on this zone [5,9].

Many studies have been conducted to characterize the microstructure of the ITZ in concrete using different tools such as scanning electron microscopy (SEM) and energy dispersive X-ray (EDX) [10,11]. These tools have been widely applied to identify the microstructural gradients through the ITZ in concrete [12,13]. However, the aforementioned techniques are limited because the local mechanical properties across the ITZ cannot be quantified using the SEM and EDX. Micro-indentation was developed to characterize the mechanical properties of different microstructural gradients at the ITZ in concrete [14,15], but the size of the nano-indent in comparison with the thickness of ITZ limits its applications for concrete. Nanoindentation, which has been widely used to study the local mechanical properties of other materials [16], has been increasingly adopted in cementitious materials [17,18,19]. One substantial advantage of nanoindentation over micro-indentation is its nano-indent size, and this advantage allows the intrinsic properties of microstructural gradients across the ITZ to be characterized. Studies such as [20,21] were the first that implemented nanoindentation for ITZ characterization. The procedure for sample preparation for the nanoindentation test was developed in [20], and the interface between fiber–matrix–aggregate was investigated. The nanoindentation was coupled with SEM-EDX and laser scanning to explore the weakest zone in concrete [21].

It is well known that the most commonly used binding agent in construction is Portland cement (PC), but its production releases a large amount of CO_2_ [22,23]. On the other hand, the use of geopolymer concrete has recently attracted interesting attention as an alternative to traditional concrete. Geopolymer emits less CO_2_ compared to traditional concrete [24,25]. Geopolymer is produced by mixing byproduct materials such as fly ash with an alkaline solution. Fly ash is the best candidate to make geopolymer, since it required less alkaline solution to be activated when compared to metakaolin [26]. Its worldwide availability is also one of the main reasons that make it the most appropriate material to produce geopolymer [27]. The reaction (geo-polymerization) in geopolymer starts with the dissolution of reactants in an alkaline solution, with resulting polymeric Si–O–Al–O bonds in the amorphous form [24,28]. The mechanical properties and durability of geopolymer concrete have been reported by many researchers to be comparable with those of traditional concrete [29,30,31,32]. The compressive strength of geopolymer was 30 MPa, while that of OPC concrete was around 27 MPa [29]. The compressive strength of geopolymer and geopolymer concrete decreased by 35% and 63%, respectively, after one year of immersion in sulfuric acid solution [29]. After exposing geopolymer and OPC specimens in 10% sulfuric acid and 10% magnesium sulphate solutions, the geopolymer presented a better resistance compared to OPC specimens [30]. Geopolymer specimens with reinforced rebars were tested under accelerated corrosion test [31]. The results showed that geopolymer specimens had good resistance to chloride attack, with a longer time before corrosion cracking compared to OPC specimens [31]. The aforementioned statement leads to the conclusion that the mechanical properties and durability of geopolymer can make it better a construction material than OPC, but there is not enough information regarding the ITZ in concrete and its properties in geopolymer concrete.

Despite the great attention given to using geopolymer concrete as an alternative to traditional concrete, only limited studies have explored the ITZ in geopolymer concrete [33,34]. With developments in technology, advanced techniques such as nanoindentation and SEM can be coupled to figure out the ITZs in geopolymer and compare this to the ITZs formed in PC concrete. Nanoindentation can be applied to quantitatively assess the nanomechanical properties of the different phases [21,33,35], while the microstructure of ITZs’ phases can be characterized by SEM [5,21,33]. In geopolymers, the main reaction product is called sodium aluminosilicate hydrate (N–A–S–H) gel, and the indentation modulus is around the indentation modulus of the main hydration of PC paste product (low-density C–S–H) [36,37,38].

The main aim of this research is to study the microstructures and nanomechanical properties of the ITZ in a geopolymer concrete mixture. Toward this end, SEM and nanoindentation tests were utilized to investigate the properties of the ITZ in geopolymer concrete at the microscale, and the results were compared with ITZ in conventional concrete.

## 2. Results and Discussion

### 2.1. Microstructure Analysis

**Figure 1:**
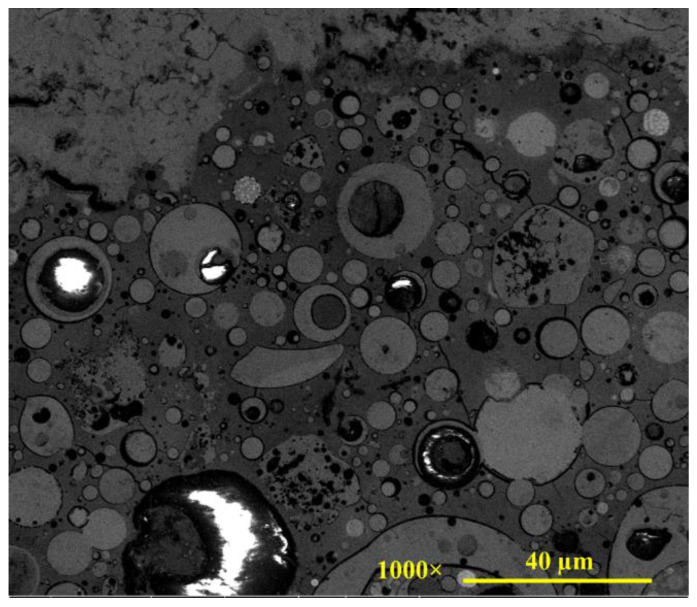
SEM image of geopolymer microstructure.

**Figure 2:**
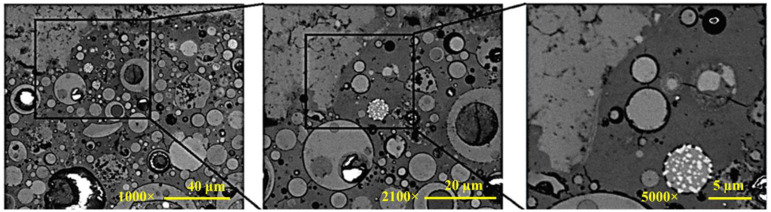
Consecutive zoomed SEM images of geopolymer mixture.

**Figure 3:**
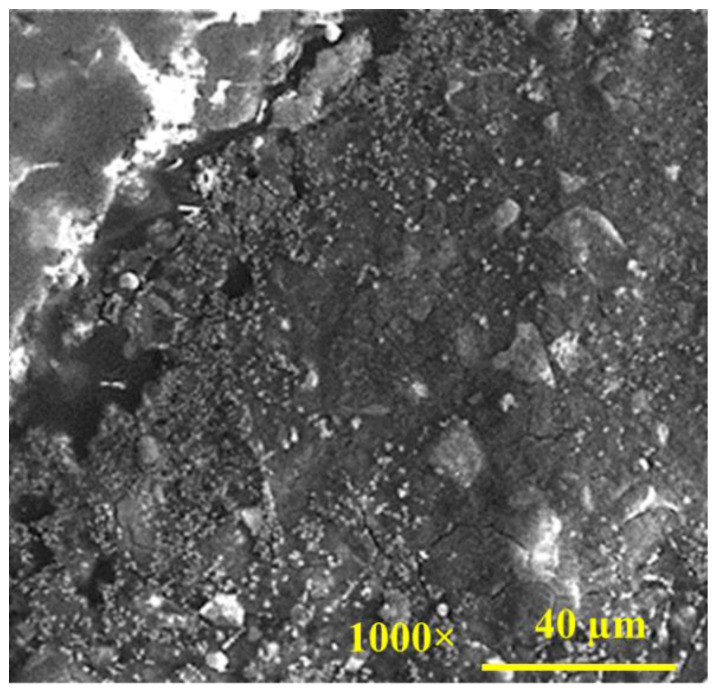
SEM image of OPC concrete microstructure.

The SEM microstructure image of the geopolymer concrete mixture is shown in Figure 1. The geopolymer mixture image clearly shows a complicated matrix microstructure with multiple phases. Thus, it is expected that the individual properties of those phases and the interactions between the phases play a significant role in the overall physical–mechanical properties of geopolymer concrete. This role is discussed in more detail later. Regarding the geometric characteristics of the ITZ formed, the microstructures in the vicinity of large aggregate (limestone) did not present clear differences from the matrix phase microstructure, without showing any obvious voids around the limestone particle. The zoomed-in images presented in Figure 2 further demonstrate the bonding that occurs at the ITZ between aggregates and the geopolymer matrix. The microcracks shown in the images, although this is not certain at this stage, may have been caused by extra stress during the polishing (surface smoothing) process or the cementitious shrinkage during the curing process. On the contrary, the SEM microstructure image of the OPC mixture shows large voids/pores around the ITZ, as seen in Figure 3. This observation agrees well with similar studies [21,39]. Low-density ITZ would compromise OPC mixture properties by weakening the entire mixture. The weak ITZ in OPC mixtures is typically related to calcium hydroxide crystals and pores concentrated at the ITZ due to rich water at the aggregate surface [40]. Such crystals were not found in geopolymer paste or ITZ.

### 2.2. Nanomechanical Properties

**Figure 4:**
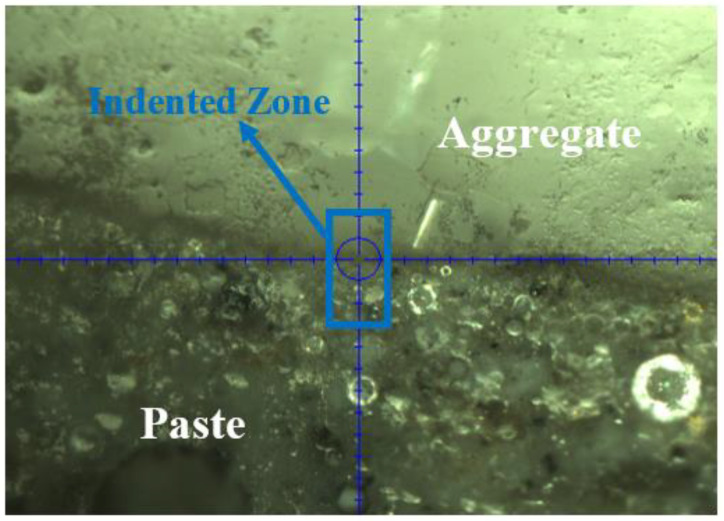
A geopolymer microstructure (780 µm by 585 µm) with multiple phases and indentation zone.

Nanoindentation tests were conducted to measure the elastic properties in the microstructures, including the ITZ region in geopolymer concrete and OPC concrete. A total distance of approximately 140 μm crossing multiple phases (from the aggregate to cementitious matrix) in the microstructures was inspected. One example is shown in Figure 4. The figure shows the testing zone across the interface for the geopolymer specimen. The distance between each nano-indent was 3 μm to avoid any interactive issues between nano-indents [41].

**Figure 5:**
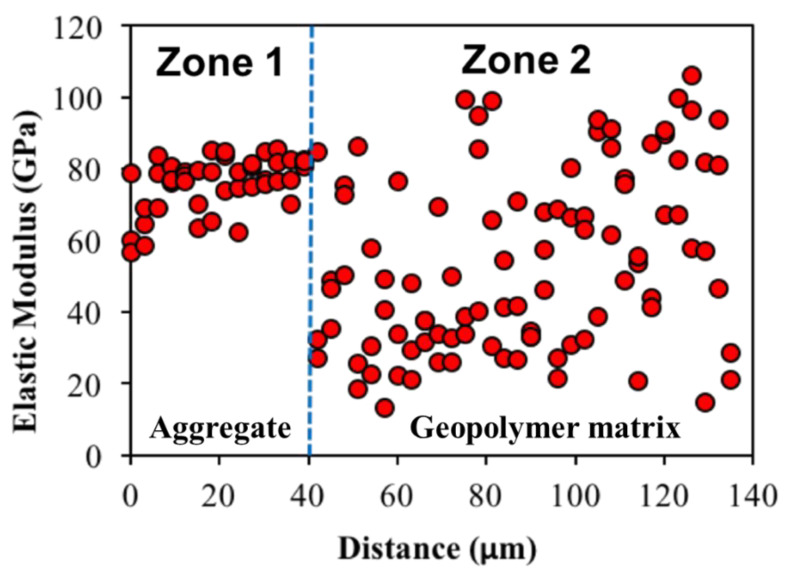
Indentation modulus over the aggregate and geopolymer paste of the geopolymer concrete.

The elastic modulus distributions of the geopolymer mixture are shown in Figure 5. The figure shows that each region, when dominated by different phases, can be recognized by the variation in the indentation modulus. Roughly, two zones were observed: the first zone, which is approximately 40–50 μm, where indentation modulus are less scattered and the second zone with indentation modulus highly scattered. The first zone is for aggregate. The zone limitation is the significant change in the indentation modulus around the aggregate surface or far from the aggregate in the matrix, which can be considered as a limitation of the zones. There was no obvious transition between the two regions. Considering the direction of the indentation pathway, test results indicate that elastic stiffness is generally consistent within the aggregate particle and becomes more scattered in the matrix phase. There is no clear weak zone identified around the aggregate with lower modulus, such as the ITZ typically observed from OPC mixtures [39,42]. This outcome can be attributed to the high bond strength of the geopolymer matrix, as presented in a recent study [43], and as the SEM images shown in Figure 2 demonstrate. Based on the SEM image shown in Figure 1, the highly scattered stiffness values within the matrix phase seem strongly related to the different compositions such as N-A-S-H gel, partially reacted fly ash particles, unreacted fly ash particles, and pores [35,37,38]. For comparison purposes on a large scale between geopolymer and OPC concrete, the compressive strengths of geopolymer and OPC were 36.2 MPa and 38.6 MPa, respectively.

**Figure 6:**
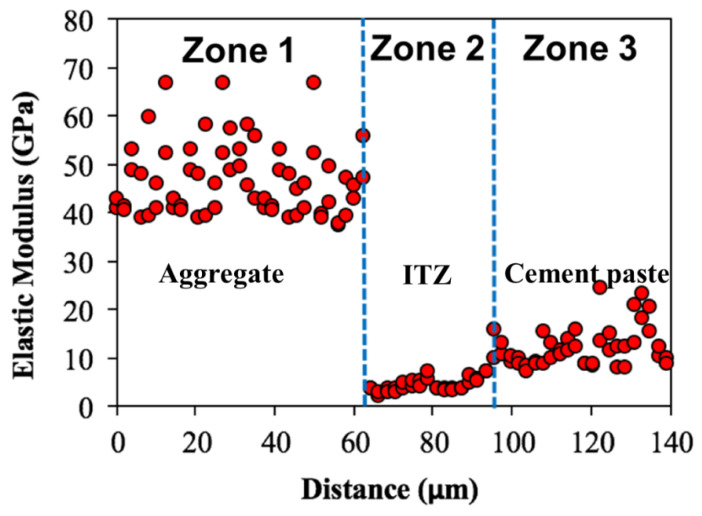
Indentation modulus over the aggregate and cement paste of the OPC concrete.

The elastic modulus distributions of the OPC mixture are shown in Figure 6. Comparing these to the results from the geopolymer case, it can be seen that the nanoindentation test results shown in the figure are quite different. Based on the findings from the nanoindentation results, three zones were generally observed, with an intermediate zone where indentation modulus is lower than in the other two zones. The first zone, which is approximately 60 μm, is seen as an aggregate particle by considering the indentation direction and the elastic modulus values obtained (i.e., average 49 GPa). The modulus dropped sharply, then increased gradually. It is implied that the intermediate region, with a width of approximately 30 μm, is the ITZ where lower density entities such as voids/pores exist as shown in Figure 3. After the ITZ zone, the distribution of indentation modulus in the cementitious matrix ranges between 10 to 30 GPa with much less scatter than the geopolymer cases. The indentation modulus of the low-density C–S–H is between 15–31 GPa [44]. This means that the microstructure of the cement paste is mostly composed of C–S–H. On the other hand, around 50% of the microstructure of geopolymer paste is composed of unreacted and partially reacted fly ash particles [33], which is in line with the findings in this study. The average indentation modulus of partially reacted and unreacted particles is 40 GPa and 70 GPa, respectively [33]. Nanoindentation results shown in Figure 4 and Figure 5 are well aligned with the SEM visual observations, which indicates that weak ITZ is not clearly detected in geopolymer concrete, whereas the weak ITZ around aggregates can be an issue in OPC mixtures. The weak ITZ in OPC has been reported in many other studies [41,42,45], and this study confirms their findings.

**Figure 7:**
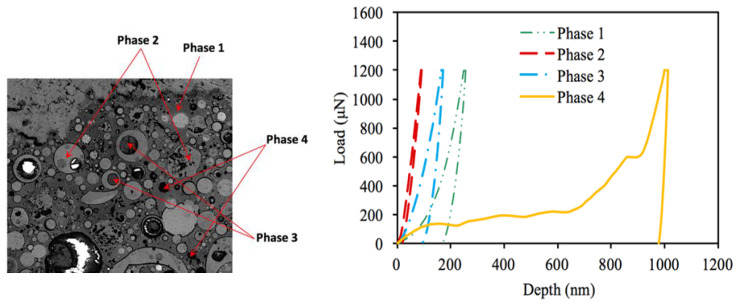
Typical nanoindentation test results of different phases in geopolymer matrix.

In addition to the sequential indentations made along the longitudinal direction across the three zones (aggregate–interphase–matrix) to investigate property changes with a particular interest in the interphase zone, the nanoindentation test was also conducted to further examine properties of individual phases observed in the geopolymer matrix. Based on the SEM images, the indentation moduli measured within the cementitious matrix can be categorized into four different major phases, as marked in Figure 7. Identification of individual phases can be better investigated by the resulting nanoindentation tests (i.e., load–depth curves and the resulting mechanical properties such as hardness and elastic modulus). Figure 7 also shows representative loading–unloading indentation curves for individual phases. As shown, the circular phase in lighter gray (Phase 2) experiences very small indentation depth resulting in the highest elastic modulus, while the small black dots (Phase 4) caused considerable indentation depth (around 1000 nm), which resulted in very low stiffness. The circular phase in darker gray (Phase 3) produced a larger indentation depth than the circular phase in lighter gray (Phase 2). The surrounding continuous phase (Phase 1) had larger indentation depths than the two circular gray phases, which resulted in lower stiffness than the two heterogeneities. Although it is not conclusive at this stage, by integrating the visual observation with nanomechanical behavior, the continuous phase is regarded as a geo-polymerization product, N–A–S–H gel, and the circular gray phases are fly ash particles that are partially reacted at different levels (more reacted is represented in darker gray). The black dots are regarded as small pores considering their large indentation depth. The pores in geopolymer paste appear in uniform spherical shapes, whereas the shape of pores in OPC paste is irregular [46,47].

## 3. Conclusions

This study examined microstructures and nanomechanical properties of geopolymer concrete mixtures where Class F fly ash is considered an alkali-activated binder. Two primary laboratory tests, SEM analysis and nanoindentation were conducted for geopolymer mixtures and an OPC concrete mixture as a counterpart of the geopolymer mixture. Based on test and analyses results, the main conclusions are drawn below:

- SEM test results show that traditional concrete developed a weak ITZ with voids and cracks, while the geopolymer concrete microstructure did not present weak ITZs in the vicinity of aggregates.
- Nanoindentation results showed that, in geopolymer mixtures, there is no clear weak zone identified around the aggregate with lower modulus such as the ITZ typically observed from OPC mixtures. This observation can be attributed to the high bond strength of the geopolymer matrix.
- Geopolymer matrix presented multiple phases in different geometries (SEM results) and mechanical properties (nanoindentation results). Highly scattered stiffness values within the matrix phase were observed and seem strongly related to the formation of different compositions such as N-A-S-H gel, partially reacted fly ash particles and pores.
- The investigation of geopolymer matrix performed in this study will provide a better understanding on performance at large scales, which could lead to enlarge the filed application of geopolymer.
- For future study, it is recommended to investigate the effect of aggregate surface texture on the bond properties between the aggregate and binding agents.

## 4. Materials and Methods

### 4.1. Materials

The binding agent used to produce geopolymer is class F fly ash with a specific gravity of 2.37. The chemical composition of fly ash is SiO_2_ = 51.80%, Al_2_O_3_ = 23.06%, Fe_2_O_3_ = 13.03%, CaO = 2.81%, MgO = 0.85%, SO_3_ = 1.23%, Na_2_O = 0.71%, and K_2_O = 2.52%. Sodium hydroxide with a molar concentration of 12 is mixed with sodium silicate to prepare the alkaline solutions used to activate the fly ash. As reported in previous studies [48], combining sodium hydroxide solution with sodium silicate solution would lead to higher geo-polymerization. The mass ratio of SiO_2_ to Na_2_O in sodium silicate solution is 3.1, and 63% of sodium silicate solution is water.

### 4.2. Samples Preparation

The preparation of geopolymer concrete specimens begins with dry mixing the fly ash with limestone for three minutes. Then, the alkaline solution is added gradually and mixed for five minutes. The ratio of sodium silicate solution to sodium hydroxide solution used in this study is 2.5. Immediately after mixing, the mixtures were cast in (100 × 200 mm cylindrical molds). The geopolymer specimens were placed in an oven at 60 °C for 24 h. After 24 h of curing in the oven, the specimens were taken out and demolded, then left for 28 days in the lab. The alkaline solution for fly ash was 0.4. OPC (Type I) concrete was also prepared with a design strength of 35 MPa in order to investigate the ITZ formation in OPC concrete and compare the results with those of geopolymer concrete. The size distribution and quantity of coarse aggregates were the same for all mixtures to ensure an effective comparison. The aggregates that were used to prepare concrete specimens were coarse aggregates that were retained on sieves of 4.75 to 12.5 mm. Fine aggregate was excluded to focus the study on the ITZs. The mix proportions are summarized in Table 1.

To prepare the specimens for nanoindentation and SEM tests from the cylinder sample (100 × 200 mm cylindrical molds), small thin sections measuring approximately 10 mm by 10 mm by 5 mm were cut with a low-speed precise diamond saw. Since the nanoindentation tests were conducted at small scales, the roughness of the specimen surfaces has a huge impact on the accuracy of the results. In order to obtain a smooth surface that is crucial for micro- and nano-level experiments, each sample cut was grounded and polished to achieve a final level of smoothness of less than 100 nm. An optical microscope was used to examine the effectiveness of the grinding and polishing procedures. Finally, specimens were cleaned in an ultrasonic bath and then kept in airtight containers.

### 4.3. Test Methods

In order to investigate the microstructure in geopolymer and OPC concrete mixtures, SEM was used on the polished surface to acquire microscale images. SEM provides images that contain significant details about the microstructure. One of the powerful SEM imaging techniques is Backscattered Electron mode. By using this technique, different phases of the sample can be identified depending on their atomic numbers, which appear at different grey levels. Differences in atomic numbers indicate the different chemical compositions of each phase.

**Figure 8:**
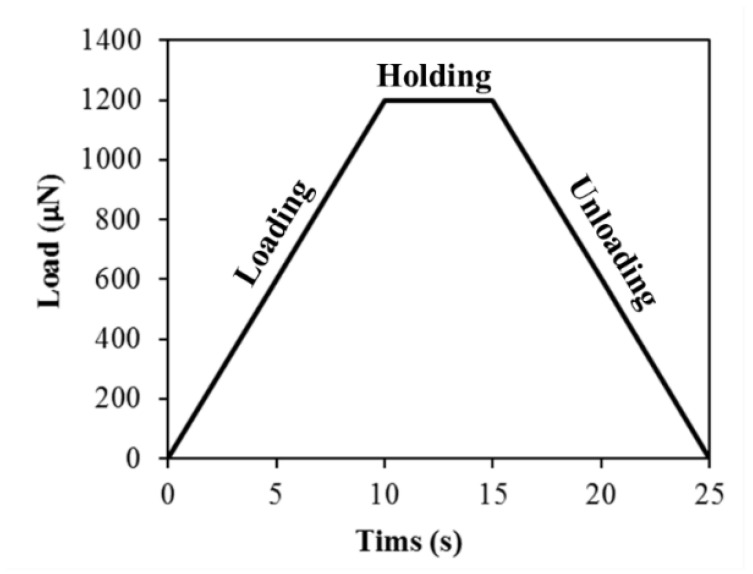
Loading diagram of the indention test.

To determine the nanomechanical properties of each specimen with a focus on the ITZ, a Hysitron Triboindentor equipped with a pyramidal-shaped diamond Berkovich tip was utilized to conduct the nanoindentation tests. Similar to these studies [49,50], the loading function was applied under load-control mode with a maximum load of 1200 µN, achieved in 10 s with a loading rate of 120 µN/s followed by five seconds holding and unloading at the same rate as shown in Figure 8. The holding for five seconds is necessary to eliminate any creep effects.

## Figures and Tables

**Table 1:** Details of mix proportions.

Mix	Portland Cement, g	Fly Ash, g	Coarse Aggregate Fraction (mm), g			Water, mL	Sodium Silicate Solution, mL	Sodium Hydroxide Solution, mL
			19–12.5	12.5–9.5	9.5–4.75			
Geopolymer	0	1000	680	567	453	0	286	114
OPC	1000	0	680	567	453	400	0	0

## Data Availability

The data presented in this study are available on request from the corresponding author.
